# American Older Adults in COVID-19 Times: Vulnerability Types, Aging Attitudes, and Emotional Responses

**DOI:** 10.3389/fpubh.2021.778084

**Published:** 2022-02-08

**Authors:** Mingqi Fu, Jing Guo, Xi Chen, Boxun Han, Farooq Ahmed, Muhammad Shahid, Qilin Zhang

**Affiliations:** ^1^Center for Social Security Studies, Wuhan University, Wuhan, China; ^2^Department of Health Policy and Management, School of Public Health, Peking University, Beijing, China; ^3^Department of Health Policy and Management, Yale School of Public Health, New Haven, CT, United States; ^4^Department of Anthropology, Quaid-i-Azam University, Islamabad, Pakistan; ^5^Department of Anthropology, University of Washington, Seattle, WA, United States; ^6^School of Insurance and Economics, University of International Business and Economics (UIBE), Beijing, China

**Keywords:** vulnerability, aging attitudes, emotion, older adults, COVID-19

## Abstract

The Coronavirus Disease (COVID) pandemic has aroused challenges to emotional well-being of the individuals. With 1,582 respondents from the Health and Retirement Survey (HRS), this study investigates the heterogeneity in older adults' vulnerability and examines the relationship between vulnerability types, aging attitudes, and emotional responses. International Positive and Negative Affect Schedule Short-form (I-PANAS-SF) and Attitudes toward own aging (ATOT) were used to assess the emotional experiences and aging attitudes, and 14 kinds of pandemic-related deprivations evaluated vulnerability of individuals. Latent class analysis (LCA) was used to explore the vulnerability types, and weighted linear regressions examined the relationship between vulnerability, aging attitudes, and emotional responses. The results showed that the proportion for individuals with mild vulnerability (MV), healthcare use vulnerability (HV), and dual vulnerability in healthcare use and financial sustainment (DVs) was 67, 22, and 11%, respectively. Older adults aged below 65, Hispanics and non-Hispanic Blacks, and those not eligible for Medicaid were more likely to have HV or DVs. The relationship between vulnerability and positive emotions (PAs) was non-significant, yet individuals with HV (beta = 0.10, standard error [SE] = 0.16) or DVs (beta = 0.09, SE = 0.28) were likely to have more negative emotions (NAs) than their mildly vulnerable counterparts. Furthermore, aging attitudes moderated the relationship between vulnerability and emotions. The salutary effect of positive aging attitudes on emotional well-being was more significant among people with DVs than those with MV (beta = 0.20, SE = 0.04 for positive responses; beta = −0.15, SE = 0.04 for negative responses). Thus, we urge more attention for vulnerable older adults in a pandemic context. Meanwhile, encouraging positive aging attitudes might be helpful for older adults to have better emotional well-being, especially for those with DVs.

## Introduction

The Coronavirus Disease (COVID-19) pandemic brought substantial mental health impacts besides direct threats to the physical health of individuals. With the influences of pandemic threats, changes in routine, worries about financial loss, and loneliness during this public health upheaval, people faced multiple challenges to emotional well-being (1). Prevailing evidence indicated that emotional distress was increased in several countries (2). According to the Strength and Vulnerability Integration Model, older adults have strength in regulating emotions with a better use of attentional strategies, appraisals, and behaviors, yet with vulnerability in modulating the high and sustained levels of physiological arousal (3). Despite older adults generally having higher levels of emotional health, they might experience heightened negative emotions (NAs) when faced with acute stresses, such as the outbreak of Severe Acute Respiratory Syndrome (SARS) (4). About one-half of older adults in the hard-hit United States reported stress related to the disease, while a quarter developed negative mental health responses (5). As emotions have a great potential in affecting life satisfaction and coping strategies, a growing body of research studies tried to explore the risk and protective factors of emotional well-being in the COVID-19 context (6, 7). Individual characteristics, such as gender, age, race, the level of education, marital status, economic status, the ability of daily activities, and self-perceived health status had been intensively examined (6, 8). However, based on our information, a few studies have examined the association between emotional well-being and vulnerability among older adults.

Vulnerability is the result of a set of risks in threat exposure, threat materialization, and lack of defense to cope with the threat (9). In previous studies, the vulnerability could be evaluated either by models targeting individuals' deficiencies in the face of hazards or by models focusing on the outcomes of these risks (10). Referring to existing studies (11–14), we measure the vulnerability during COVID-19 with the risks in pandemic-related deprivations, which are rooted in older adults' deficiencies in disease prevention, healthcare utilization, financial resilience, and housekeeping capability. More detailed, in the face of highly infectious disease, declining immune function among older adults, and defective preventive strategies of their families may put individuals at increased risk of infection (11). Besides, existing studies suggest that older adults are more likely to have inadequate healthcare use and financial hardships than their younger counterparts in emergent circumstances (15–18). Moreover, they might have to deal with overwhelming chores during the pandemic because of austere hygiene challenges and disruption in housekeeping service (19). However, the vulnerability of older adults might be heterogeneous due to differential types and volumes of resources that individuals possess. People may have different levels of risk to experience the pandemic-related deprivations and might be distinguished as having single-dimensional or multiple-dimensional vulnerability. However, most existing COVID-19-related studies treated the vulnerability of older adults homogeneously (7, 20); to date, none of them have revealed the heterogeneity in the vulnerability of the older adult population.

Previous studies proposed that vulnerability during adverse events might arouse negative emotional responses (4, 21). However, researchers have reached no consensus regarding the association between vulnerability and positive emotional responses. In some studies, positive emotions (PAs) were decreased when the person had a severer vulnerability (22, 23). By contrast, other studies found no significant reduction in PAs despite the vulnerability of older adults (24, 25). Besides differences in trauma types, variation in the affective profiles of the respondents also contributed to this inconsistency. The affective profile is a psychological trait with an orthogonal structure describing individuals prone to PAs and NAs in the face of life challenges (26). Accordingly, affective profile of an individual may involve four main types: self-fulfilling, low-affective, high affective, and self-destructive. People with a self-fulfilling profile are often more energetic and optimistic and perform better in maintaining emotional well-being than the other three affective types in stressful situations (27). In addition, emotional responses during an adverse event are linked with the rumination on pandemic-related deprivations, as the Cognitive Appraisal Theory of Emotion was noted (28). In unintentional situations, positive internal schema and PAs would sustain when older adults attribute their vulnerability to external factors. Alternatively, considering the vulnerability as a result of personal inability may lead to self-depreciation and expel PAs. Nevertheless, during the COVID-19 pandemic, how older adults conceive their vulnerability may be differential across populations (14). Thus, in the current study, we examine the relationship of vulnerability with positive and negative emotional responses, thereby evaluating the emotional well-being of older adults amidst the COVID-19 threats.

Moreover, aging attitude as a cognitive pattern might moderate the relationship between vulnerability and emotional responses. The Cognitive Vulnerability-Stress Model suggests that older adults with positive aging attitudes would have better subjective well-being during adverse events (29). When confronted with risks in pandemic-related deprivations, positive aging attitudes would improve the emotional well-being *via* emotional and informational processing. On the one hand, self-esteem from positive aging attitudes serves as a shield against initial negative reactions, which is helpful to restrain stress-diathesis and have better use of emotion regulation strategies (30). On the other hand, positive aging attitudes would help individuals to effectively select the pandemic-related information (31). People with positive views on aging are less likely to be impacted by discriminative information against older adults, thereby conducting fewer negative ruminations (32). However, whether the association between positive aging attitudes and emotional responses vary between vulnerability types remains unclear. Theoretically, older adults experiencing fewer dimensions of insecurities may have greater self-esteem and confidence to overcome the pandemic after comparisons with their multiple-dimensional damaged counterparts (33). Such a sense of capability is intrinsically inherent with positive aging attitudes and might amplify their salutary effects on the emotional well-being. Alternatively, positive aging attitudes might also be more important for populations with multiple-dimensional vulnerability, helping to maintain positive self-images under substantial pandemic-related deprivations (34). Given the debates above, this study tries to examine the interactive effect of vulnerability and aging attitudes on the emotional well-being of older adults.

### Aims and Hypotheses

This study investigates the latent vulnerability types among American older adults and examines the relationship between vulnerability type, aging attitudes, and emotional responses in COVID-19 settings. Our first hypothesis concerns the latent vulnerability types with an investigation on disease infection, delayed healthcare use, financial hardships, and overwhelming chores. This hypothesis is exploratory, assuming older adults might be distinct as having a single-dimensional or multiple-dimensional vulnerability due to differential types and volumes of resources they possess. Moreover, the socioeconomic and health characteristics of vulnerability groups would be different. Secondly, we propose that multiple-dimensional vulnerability is associated with higher levels of negative emotional responses. In contrast, the relationship between vulnerability and positive emotional responses might be negative or non-significant. Lastly, we assume that older adults with positive aging attitudes would have more positive and fewer negative emotional responses. Meanwhile, positive aging attitudes might moderate the relationship between vulnerability and emotional responses. The salutary effect of positive aging attitudes on emotional well-being might be more significant for individuals being either single- or multiple-dimensional vulnerable in the pandemic.

## Materials and Methods

### Study Design and Data Collection

Health and Retirement Survey (HRS) is a national longitudinal study of older Americans' health and economic situation (https://hrsonline.isr.umich.edu/). Data in this study were used from the 2020 HRS COVID-19 Project (Early, version 1.0). The COVID-19 module administrated 50% random subsample of households initially assigned to enhance the interviewing. This 50% random subsample was further split into two random samples: the first one was released to fieldwork on June 11, 2020, while the second one was on September 24, 2020. Information in this study was gained from the first random sample of 3,266 respondents, accounting for ~25% of the original HRS sample. Due to lockdowns in the pandemic, the COVID-19 Project was conducted via telephone, with a response rate of 62%. Detailed information on sampling design, survey content, and sample weights of HRS can be found elsewhere (35). After excluding persons below age 60 and who did not report emotional responses, this study included a total of 1,582 cases. All regressions were weighted using inverse probability weights to adjust for selections and non-response in the data.

The Institutional Review Board (IRB) of the University of Michigan approved the HRS survey, while the IRB of Yale University provided approval for this study. All methods were performed in accordance with the Declaration of Helsinki. As some of the older adults could not write, all respondents provided verbal consent to this survey.

### Measures

#### Outcome Variable

Emotional responses in this study were measured with the International Positive and Negative Affect Schedule Short-form (I-PANAS-SF) (36), which includes 10 items and estimates the degree of PAs and NAs that individuals experienced in the past month. Five positive emotional responses involved active, determined, attentive, inspired, and alert, whereas five negative responses included afraid, nervous, upset, hostile, and ashamed. Older adults were invited to rate these emotional responses on a 5-point scale according to the extent to which they have felt, while higher scores referred to more intensive affectivities. The I-PANAS-SF is psychometrically acceptable across cultures (37). In the current study, the internal consistency coefficient (Cronbach's α) for PAs and NAs was 0.811, 0.776, respectively, indicating acceptable reliability of the measurement.

#### Independent Variables

The vulnerability was assessed with 14 items of pandemic-related deprivations rooted in older adults' deficiencies in disease prevention, healthcare utilization, financial resilience, and housekeeping capability. With references from the Sensitivity and Resilience Model and related empirical studies (11–14), this study made use of the most typical symptoms for each dimension. Disease infection of individuals and their family members exhibited older adults' vulnerability in disease prevention. Delay for surgery, prescription filling, doctor visits, dental care, and other services expressed vulnerability in healthcare utilization. Experiences of income deduction, spending growth, food shortage, missing financial dues, asking help with bills, and other hardships showed vulnerability of the individuals in financial resilience. Then, asking for help to do chores represented a vulnerability in housekeeping capacity. We invited older adults to report if they had experienced each of the 14 items during the pandemic (since April 2020), with 0 refers to no and 1 for yes. The vulnerability was calculated as a categorical variable after latent class analyses (LCA), with 0 for mild vulnerability (MV), 1 for healthcare use vulnerability (HV), and 2 for dual vulnerability in healthcare use and financial sustainment (DVs).

Aging attitudes were examined with a brief five-item unidimensional measure that compromises the Expansion: Attitudes towards own aging (ATOA) dimensions of the Philadelphia Geriatric Center (PGC) Morale Scale (38). Items from the ATOA measure included: “I have much pep as I did last year,” “I am as happy now as when I was younger,” “Things keep getting worse as I get older,” “The older I get, the more useless I feel,” and “As I get older, things are better than I thought they would be.” A 6-point response scale was used to evaluate the degree of each item. When negative items were reverse-scored, this scale captured older adults' global positive evaluation of their aging process (39). The Cronbach's α for the scale was 0.773 in this study.

#### Covariates

Affective profile was determined by the mean scores of positive affections and negative affections, reported throughout 2002–2018 HRS waves. In line with previous studies (26, 40), we adopted a cutoff point at 53.2% for PAs and 48.9% for NAs to identify the orthogonal structure of individuals' affections: self-fulfilling profile (high scores in PAs but low scores in NAs); low affective profile (low scores both in PAs and NAs); high affective profile (high scores both in PAs and NAs); and self-destructive profile (low scores in PAs but high scores in NAs). Other covariates in the current study comprised sex (male/female), age (below 65/65 and older), race (Hispanic/non-Hispanic White/non-Hispanic Black/others), marital status (married or partnered/uncoupled), educational level (less than high school/high school or above), household wealth (relatively poor/mid-level/rich), Medicaid eligibility (eligible/not eligible), difficulty in daily activities (none/one and more, i.e., 9 items, such as dressing, bathing, preparing hot meals, shopping for groceries, and so on), and self-reported health status (relatively poor/relatively good). These variables have been examined to associate with the emotional well-being of older adults in the United States (6, 8, 41).

### Statistical Analyses

Descriptive analyses were conducted for the outcome variable, independent variables, and covariates. We used Mplus Version 7 to conduct LCA to identify unobserved clusters of individuals that respond to measured vulnerability items with a similar pattern. In this stage, robust maximum likelihood (MLR) estimators were adopted. Indicators, such as Akaike Information Criterion (AIC), Bayesian Information Criterion (BIC), sample size-adjusted BIC (ssaBIC), entropy, values of the Lo-Mendell-Rueben Test (LMRT), and the Bootstrap Likelihood Ratio Test (BLRT) were used for model selection. Meanwhile, we conducted bivariate analyses between the vulnerability type and socioeconomic/health variables while considering uncertainty in membership assignment (42). In the next step, class membership was assigned to each individual based on the probability and was treated as an observed variable. We used multinomial logit regressions to examine the factors associated with the vulnerability type, of which the relative risk ratio (RRR) and 95% CI were reported. In addition, Linear regressions were conducted to examine the association between the vulnerability type, aging attitudes, and emotional responses, after adjusting for a broad spectrum of covariates, such as affective profile, sex, age, race, marital status, education, household wealth, Medicaid eligibility, difficulty in daily activities, and self-reported health. Here, standardized and unstandardized coefficients, robust SE, and 95% CI were reported. All regressions were weighted using inverse probability weights, with sample weights that have corrections for emotion non-response. List-wise deletion was used to handle missing data, and all regressions were conducted in Stata Version.

## Results

Table 1 presents the descriptive characteristics of variables in this study. Among 1,582 respondents, women (*N* = 919, 58.20%) and persons aged 65 years and older (*N* = 1,129, 71.50%) were accounted for the majority. Additionally, more than half of the respondents were non-Hispanic Whites (*N* = 1,085, 68.71%), married or partnered (*N* = 971, 61.50%), and had a high school degree or above (*N* = 1,327, 83.98%). Although 64.91% (*N* = 1,025) of the elderly had difficulty in daily activities, the proportion for self-reported poor health was only 38.53% (*N* = 608). In line with the high proportion of the self-fulfilling affective profile (*N* = 1,197, 81.93%), older adults in this study reported a relatively high level of PAs (mean = 12.60, SD = 4.21, range 0–20). By contrast, the mean score for NAs was only 3.09 (SD = 2.90, range 0–20). With regard to vulnerability items, over 20% of respondents (*N* = 336, 21.24%) lived with stressful chores and had to ask for help. Meanwhile, the prevalence for a delayed doctor visit and inadequate dental care was 16.31% (*N* = 258) and 20.35% (*N* = 322), respectively. Noteworthy, the most prevalent financial insecurities among older adults were income deduction (N = 214, 13.53%), spending growth (*N* = 277, 17.51%), and asking others to pay the bills (*N* = 304, 19.22%). Details are shown in Table 1.

**Table 1:** Descriptive analysis of the sample (*N* = 1,582).

**Characteristics**	**Mean (*N*)**	**SD (%)**	**Range**
**Emotional responses**			
Positive emotions	12.60	4.21	[0,20]
Negative emotions	3.09	2.90	[0,20]
**Aging attitudes**	19.92	5.40	[5,30]
**Affective profile**			
Self-fulfilling	1,197	81.93	
High-affective	13	0.89	
Low-affective	232	15.88	
Self-destructive	19	1.30	
**Sex**			
Male	660	41.80	
Female	919	58.20	
**Age**			
<65	450	28.50	
≥65	1,129	71.50	
**Race**			
Hispanic	183	11.59	
Non-Hispanic White	1,085	68.71	
Non-Hispanic Black	261	16.53	
Others	90	3.17	
**Marital status**			
Married or partnered	971	61.50	
Uncoupled	608	38.50	
**Education level**			
Less than high school	255	16.12	
High school or above	1327	83.98	
**Household wealth**			
Relatively poor	777	49.24	
Medium level	486	30.80	
Relatively rich	315	19.96	
**Medicaid eligibility**			
Not eligible	1,431	90.46	
Eligible	151	9.54	
**Difficulty in daily activities**			
None	554	35.09	
One or more	1,025	64.91	
**Self-rate health status**			
Relatively poor	608	38.53	
Relatively good	970	61.47	
**Vulnerability items**			
Diagnosed with the COVID-19	22	1.39	
HH member diagnosed	23	1.45	
Delayed surgery	63	3.98	
Delayed prescription filling	19	1.20	
Delayed doctor visit	258	16.31	
Delayed dental care	322	20.35	
Other delayed health services	102	6.45	
Income deduction	214	13.53	
Spending growth	277	17.51	
Food shortage due to financial hardships	74	4.68	
Missed financial dues	80	5.06	
Other financial hardships	82	5.18	
Ask sb outsides HH to help with bills	304	19.22	
Ask sb outsides HH to help with chores	336	21.24	

*SD, Standard Deviation; HH, Household*.

Table 2 compares the fit indices of four LCA models, from the 1-class model to the 4-class model, and decides on the 3-class model as the best. Based on the estimated probability of respondents from each latent class answering yes to vulnerability items, we further summarized the pattern of detected types as MV (including 67% respondents), HV (22% respondents), and DVs (11% respondents).

**Table 2:** Comparing models with different latent classes: fit indices (no. of Obs = 1,582).

**No. of groups**	**loglikelihood**	**AIC**	**BIC**	**ssaBIC**	**Entropy**	**LMR**		**BLRT**		**Proportion in class**			
						**2LL**	***P***	**2LL**	***P***	**1**	**2**	**3**	**4**
1	−4915.313	9872.627	9976.953	9910.253									
2	−4645.753	9377.505	9591.125	9454.550	0.80	535.627	<0.001	539.121	<0.001	0.24	0.76		
3	−4587.773	9305.545	9528.459	9422.008	0.80	115.208	0.034	115.960	<0.001	0.22	0.11	0.67	
4	−4557.761	9289.523	9721.731	9445.404	0.69	59.634	0.089	60.023	0.083	0.68	0.02	0.08	0.22

*Notes: Selection Criteria: Model selection starts from one latent class and should stop if (a) the AIC/BIC/ssaBIC begins to grow with another new group added; or (b) the p for LMR/BLRT turned non-significant with another new group added (p > 0.05). A model with an entropy of 0.8 or over is acceptable*.

*ssaBIC, sample-size adjusted BIC; LMR, Lo-Mendell-Rubin test; BLRT, bootstrapped likelihood ratio test*.

**Figure 1:**
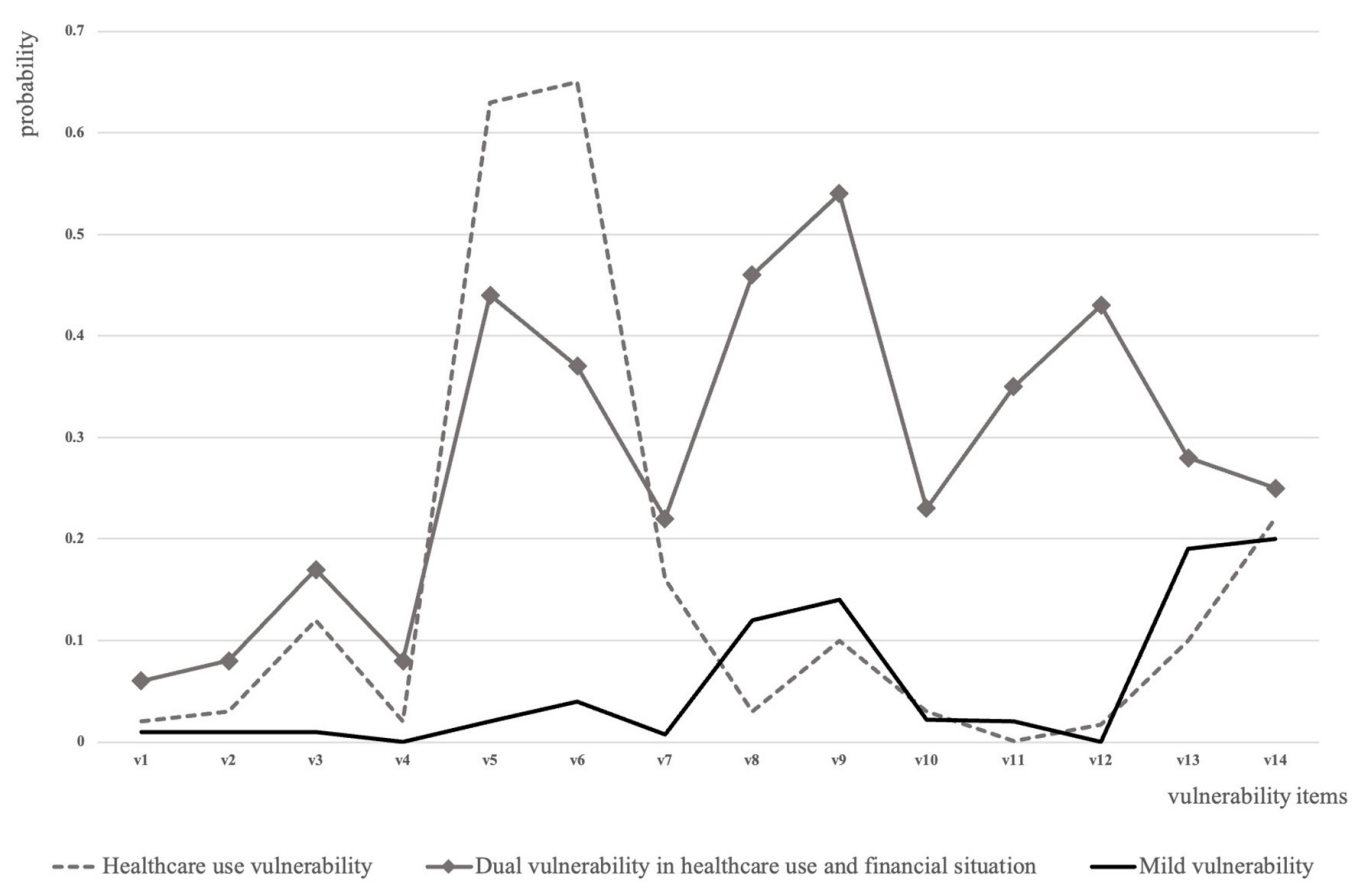
Vulnerability groups detected among older adults in the United States during the COVID-19 pandemic. Notes: The vulnerability type was determined by the estimated probability of respondents from each latent class answering yes to vulnerability items. The solid black line refers to the group with mild vulnerability; the dotted line refers to the group with healthcare use vulnerability; and the solid line with square marks refers to the group with dual vulnerability in healthcare use and financial sustainment. Vulnerability items are as follows: v1: had been diagnosed with the COVID-19; v2: had a household member been diagnosed with COVID-19; v3: had delayed surgery; v4: had delayed prescription filling; v5: had delayed doctor visit; v6: had delayed dental care; v7: had other delayed health services; v8: had income deduction; v9: had spending growth; v10: had food shortage due to financial hardships; v11: had missed financial dues; v12: had other financial hardships; v13: had to ask someone outsides household to help with bills; and v14: had to ask someone outsides household to help with chores.

As shown in Figure 1, the probability of individuals with MV to endorse pandemic-related deprivations is almost zero except 12% for income deduction, 14% for spending growth, 19% for requesting help with bills, and 20% for requesting help with chores. Distinct from mildly vulnerable persons, older adults with HV were with significantly higher risks in delayed healthcare utilization, whose probability of experiencing delayed doctor visits and dental care were both over 60%. However, the chance for individuals from the HV group to have financial hardships was all below 10%. Meanwhile, the DVs group simultaneously demonstrated significant risks in healthcare use and financial sustainment. Apart from a 22–54% chance of having items of financial hardship, older adults with DVs had a 44% chance for delayed doctor visits and a 37% chance for delayed dental care.

Supplementary Table S1, Table 3 further examine the association between the vulnerability type and socioeconomic and health factors. While considering the uncertainty of membership assignment, bivariate regressions indicate that sex, marital status, age, difficulty in daily activities, affective profile, and race were significantly associated with older adults' vulnerability type (see Supplementary Table S1 for more information). With weighted multinomial regression, Table 3 demonstrates that individuals aged below 65 were more likely to have HV (RRR = 1.51, 95% CI: 1.07, 2.11) or DVs (RRR = 1.95, 95% CI: 1.05, 3.61) during the pandemic. Compared with those not eligible for Medicaid, older adults with Medicaid were less likely to have HV (RRR = 0.49, 95% CI: 0.26, 0.93). Meanwhile, Hispanics (RRR = 3.99, 95% CI: 1.89, 8.41) and non-Hispanic Blacks (RRR = 2.06, 95% CI: 1.07, 3.98) had a significantly higher risk of developing DVs. Details are presented in Table 3.

**Table 3:** Multinomial logit regression examining differences in risk factors across three detected latent vulnerability groups (reference group: MV, *N* = 1,539).

	**HV**		**DVs**	
	**RRR**	**95%CI**	**RRR**	**95%CI**
**Age** (ref: ≥ 65)				
<65	1.51^*^	(1.07, 2.11)	1.95^*^	(1.05, 3.61)
**Race** (ref: non-Hispanic White)				
Hispanic	0.70	(0.36, 1.36)	3.99^***^	(1.89, 8.41)
Non-Hispanic Black	0.68	(0.43, 1.10)	2.06^*^	(1.07, 3.98)
Others	0.48	(0.21, 1.09)	3.12	(0.84,11.60)
**Medicaid eligibility** (ref: not eligible)				
Eligible	0.49^*^	(0.26, 0.93)	1.04	(0.48, 2.26)

*Notes: Regressions were weighted, with sample weights that have corrections for emotion non-response using inverse probability weights. Non-significant characteristics, such as affective profile, gender, marital status, education, household wealth, difficulty in daily activities, and self-rate health were not presented due to space limits*.

MV, mild vulnerability; HV, healthcare use vulnerability; DVs, dual vulnerability in healthcare use and financial sustainment; RRR, relative-risk ratio; CI, confidence interval; ADL, activities of daily living; p < 0.05^*^, p < 0.001^***^.

In Table 4, the relationships between vulnerability type, aging attitudes, and positive emotional responses during the COVID-19 pandemic are presented. With covariates controlled, positive attitudes toward aging were associated with a higher level of positive emotional responses among older adults (B = 0.26, beta = 0.35, 95% CI: 0.22, 0.31). Despite that Model 2 suggests no significant difference in PAs across three vulnerability groups, we found the association between positive aging attitudes and greater PAs was more significant among individuals with DVs than their mildly vulnerable counterparts (B = 0.09, beta = 0.20, 95% CI: 0.01, 0.18).

**Table 4:** Weighted linear regressions of the relationship between aging attitudes, vulnerability type, and positive emotional responses among older adults (*N* = 1,539).

**Variables**	**Model 1**			**Model 2**			**Model 3**		
	**B (Robust S.E.)**	**95%CI**	**Beta**	**B (Robust S.E)**	**95%CI**	**Beta**	**B (Robust S.E)**	**95%CI**	**Beta**
**Aging attitudes**	0.26 (0.02)	(0.22, 0.31)	0.35^***^	0.27 (0.02)	(0.22, 0.31)	0.36^***^	0.25 (0.02)	(0.20, 0.29)	0.33^***^
**Vulnerability (ref: MV)**									
HV				−0.01 (0.25)	(−0.48, 0.48)	−0.01	−1.85 (0.95)	(−3.72, 0.02)	−0.19
DVs				0.56 (0.44)	(−0.34, 1.46)	0.03	0.54 (1.26)	(−1.94, 3.02)	0.03
Interaction (ref: AA^*^MV)									
AA ^*^ HV							−0.01 (0.07)	(−0.14, 0.13)	−0.02
AA ^*^ DVs							0.09 (0.04)	(0.01, 0.18)	0.20^*^
Constant	10.53 (1.04)	(8.50, 12.57)		10.51 (1.04)	(8.48, 12.55)		10.83 (1.04)	(8.79, 12.87)	
Covariates		Yes			Yes			Yes	

*Notes: Linear regressions were conducted with positive emotional responses as the outcome variable. All regression models controlled for affective profile, sex, marital status, age, education, race, difficulty in daily activities, self-rated health status, eligibility for Medicaid, and household wealth. Regressions were weighted, with sample weights that have corrections for emotion non-response using inverse probability weights*.

*MV, mild vulnerability; HV, healthcare use vulnerability; DVs, dual vulnerability in healthcare use and financial sustainment; AA, aging attitudes; B, coefficient; S.E., standard error; CI, confidence interval*,

* *p < 0.05*;

*** *p < 0.001*.

Table 5 focuses on the negative emotional responses among older adults, investigating its relationship with aging attitudes and the vulnerability type. After adjusting for covariates, positive aging attitudes were associated with fewer negative emotional responses (B = −0.20, beta = −0.36, 95% CI = −0.23, −0.17). Compared with mildly vulnerable older adults, persons with HV (B = 0.74, beta = 0.10, 95% CI = 0.43, 1.06) or DVs (B = 1.05, beta = 0.09, 95% CI = 0.50, 1.59) were more likely to develop NAs. Moreover, the salutary effect of positive aging attitudes in reducing negative emotional responses was significantly stronger for older adults with DVs than those with MV (B = −0.10, beta = −0.15, 95% CI = −0.19, −0.01).

**Table 5:** Weighted linear regressions of the relationship between aging attitudes, vulnerability type, and negative emotional responses among older adults (*N* = 1,539).

**Variables**	**Model 1**			**Model 2**			**Model 3**		
	**B (Robust S.E.)**	**95%CI**	**Beta**	**B (Robust S.E)**	**95%CI**	**Beta**	**B (Robust S.E)**	**95%CI**	**Beta**
**Aging attitudes**	−0.20 (0.01)	(−0.23, −0.17)	−0.36^***^	−0.19 (0.01)	(−0.22, −0.17)	−0.35^***^	−0.19 (0.02)	(−0.22, −0.15)	−0.33^***^
**Vulnerability (ref: MV)**
HV				0.74 (0.16)	(0.43, 1.06)	0.10^***^	0.88 (0.63)	(-0.35, 2.11)	0.12
DVs				1.05 (0.28)	(0.50, 1.59)	0.09^***^	2.83 (0.85)	(1.15, 4.50)	0.23^***^
**Interaction (ref: AA** ^*^MV)
AA ^*^ HV							−0.01 (0.03)	(−0.07, 0.05)	−0.02
AA ^*^ DVs							−0.10 (0.04)	(−0.19, −0.01)	−0.15^*^
Constant	11.30 (0.68)	(9.96, 12.64)		10.78 (0.69)	(9.43, 12.12)		10.40 (0.72)	(8.99, 11.81)	
Covariates		Yes			Yes			Yes	

*Notes: Linear regressions were conducted with negative emotional responses as the outcome variable. Both regression models controlled for affective profile, sex, marital status, age, education, race, difficulty in daily activities, self-rated health status, eligibility for Medicaid, and household wealth. Regressions were weighted, with sample weights that have corrections for emotion non-response using inverse probability weights*.

*MV, mild vulnerability; HV, healthcare use vulnerability; DVs, dual vulnerability in healthcare use and financial sustainment; AA, aging attitudes; B, coefficient; S.E., standard error; CI, confidence interval*,

* *p < 0.05*;

*** *p < 0.001*.

## Discussion

The COVID-19 pandemic has put older adults at increased risk of pandemic-related deprivations and negative emotional responses. This study reveals that older adults demonstrated three vulnerability types in the face of COVID-19 threats. The proportion of individuals with MV, HV, and DVs was 67, 22, and 11%, respectively. We found no significant difference in PAs between vulnerability types, after controlling for a broad spectrum of covariates, such as affective profile, sex, marital status, age, education, race, difficulty in daily activities, self-rated health, eligibility for Medicaid, and household wealth. However, older adults with HV or dual vulnerability were likely to have more NAs than their mildly vulnerable counterparts. In addition, positive aging attitudes were associated with better emotional well-being among older adults, and this salutary effect was more significant for individuals with dual vulnerability. Several findings of this study warrant further discussion.

First, we propose that older adults in the United States demonstrated three distinct vulnerability types during the COVID-19 pandemic. Even for people with MV, there was still a certain probability of asking for help with bills and chores. Consistent with one previous study (19), older adults with inadequate housekeeping capacity are likely to experience disrupted housekeeping services during the pandemic, thus having to request help from others with chores. Also, the risk of requesting help with bills is understandable. A previous study suggested that the compensation of employees and social benefits from the government were severely affected by COVID-19-induced restrictions (43), which are two of the most important income contributors for older adults (44). Besides the risk of stressful chores, individuals with HV demonstrated a significantly higher risk of endorsing delayed healthcare services. Theoretically, individuals with HV are more likely from states and counties endorsing the stay-at-home order, which restricts interpersonal contacts and suggests cancellation of elective care (14). Especially in hard-hit areas where health resources are massively reassigned for pandemic control, it becomes even more challenging for older adults to access non-infectious healthcare (45). Moreover, more than one in ten older adults had DVs. Possibly, individuals with dual vulnerability are those with lower socioeconomic status. On the one hand, one prior study noted that about half of the disadvantaged older adults in the United States are living without emergency savings, thereby with a significantly higher chance of experiencing financial hardships during recessions (46). Moreover, they are more likely to have increased expenditure as many of the mechanisms for navigating life on a limited budget became difficult during the pandemic (47). On the other hand, disadvantaged older adults are more likely to depend on public transportation to get healthcare services, which is at least inconvenient under the pandemic restrictions (48). Thus, we urge local governments and communities to keep a watchful eye on older adults during crises. Noteworthy, the vulnerability is more likely to be heterogeneous rather than homogenous, and a careful evaluation for older adults of their risks in trauma-related deprivations is critical before social services are conducted.

In addition, we suggest that older adults under 65 years were less likely to be mildly vulnerable, while those not eligible for Medicaid were more likely to have healthcare vulnerability, and Hispanics/non-Hispanic Blacks were prone to have dual vulnerability. In contrast with previous studies suggesting age as a risk factor of vulnerability (12, 13), we suggest that individuals aged 60–65 years were more likely to have financial hardships and inadequate healthcare services than their older counterparts. Two explanations rationalize this finding. Firstly, older adults below 65 years are more likely to be active in the labor market before the pandemic. However, they would find it difficult to reenter the workforce during the post-pandemic recession, thereby having a greater chance to experience financial hardships (1). Secondly, it is possible that individuals of older ages would ignore their physical discomforts and require lesser care and services, as they tend to have better subjective well-being and be more satisfied with life (49). Moreover, an existing study suggested that adults aged over 65 years conduct more telemedicine visits than their 55–65 years counterparts, thus at a lower risk of delaying healthcare (50). Apart from age, we also found that individuals not eligible for Medicaid are more likely to have healthcare vulnerability, as Medicaid promotes healthcare access during the pandemic (51). Furthermore, we found Hispanics and non-Hispanic Blacks are more likely to have dual vulnerability than their Whites counterparts. One recent review suggested that Hispanics and non-Hispanic Blacks experienced higher rates of infection, hospitalization, and mortality in the pandemic (52). According to Blumenshine's model (48), Hispanics and non-Hispanic Blacks depend largely on public transportation and have limited capacity to work at home, thus becoming harder to access healthcare services and sustain income during the pandemic. In a vicious cycle, they have a greater risk of infection to get a job or seek healthcare services, which intensifies the probability of inadequate healthcare use and financial hardships once individuals become infected. In a nutshell, we propose that older adults below 65 years, being Hispanics or non-Hispanic Blacks, and not eligible for Medicaid are more likely to have healthcare vulnerability or dual vulnerability during the pandemic, which is worthy of more care and services.

Third, this study reveals that the vulnerability type of older adults presented no significant relationship with PAs but was significantly associated with NAs during the pandemic. Previous evidence claimed that severe vulnerability would lead to stress and a ruined sense of self-continuity, thus reducing PAs in young adults (53). However, this study found no significant difference in PAs between older adults from different vulnerability groups. Possibly, intrinsic motivation for emotionally meaningful goals among older adults could rationalize this finding, which is examined to be helpful to sustain PAs under challenging situations (54). Often, older adults are prone to pay attention to positive stimuli over negative information during stressful events, thus being easier to develop PAs (55). However, emotional regulation strategies are harder to work on negative arousals under prolonged stress (56). With the introduction of the sense of relative deprivation (57), people with healthcare vulnerability or dual vulnerability are understandable to have a higher level of NAs. Individuals in traumatic events are prone to compare their vulnerability with others. Nevertheless, compared with mildly vulnerable counterparts, older adults with healthcare or dual vulnerability might have a sense of unfairness, which is likely to activate negative responses such as hostility and anger (58). Thus, although older adults are often more resilient in emotional well-being, their NAs should also be noted and timely intervened when individuals demonstrate healthcare or dual vulnerability in crises.

Lastly, we propose that positive aging attitudes benefited older adults' emotional well-being in the COVID-19 context, especially for individuals with DVs. As noted by prior studies, older adults have endured prevalent discrimination during the pandemic, as some young adults may blame the dramatic response of COVID-19 as an “old people problem” (59, 60). Tags, such as #BoomerRemover#, are prevalently endorsed to express the hostility toward the elderly, exacerbating social discrimination toward older adults in the pandemic settings. Often, old persons may have emotional exhaustion under ageism discrimination, yet positive aging attitudes help to promote self-appreciation and thus against negative ruminations (61). In particular, positive attitudes toward aging might be even more critical for older adults with dual vulnerability. As noted by the attribution theory (62), older adults with multiple-dimensional vulnerability are more likely to make internal attributions for their pandemic-related deprivations. Often, self-blame is associated with greater internal ageism (i.e., people feeling ashamed for their age) and more negative affections among older adults (63). However, positive aging attitudes might alleviate such internal ageism via informational and behavioral processing. On the one hand, positive aging attitudes help individuals avoid ageism information and conduct fewer negative ruminations on that (64). Also, older adults with positive aging attitudes are less likely to consider their vulnerability during the pandemic as a threat to future lives, which might lower prospective fear and worries (65). On the other hand, people with positive aging attitudes are more likely to conduct adaptative behaviors to cope with their vulnerability during the pandemic, which establishes a better sense of self-efficacy and would mitigate the internal ageism (66). Therefore, we suggest that encouraging positive aging attitudes might be a critical approach for social services to promote emotional well-being for older adults, especially those with dual vulnerability.

### Strengths and Limitations

This study is among the first to explore the heterogeneity in vulnerability of the older adults during the COVID-19 pandemic. We provided novel evidence to reveal three latent groups of vulnerability and the relationship between vulnerability type, aging attitudes, and emotional responses. However, some limitations of this study should be acknowledged. First, this study reports that 1.39% of respondents were diagnosed. The lack of COVID-19 tests, especially at the beginning of the pandemic, and the potential stigma associated with the infection may contribute to the underestimation of the infection rate in this sample. Given the overstretched healthcare system and financial needs (before Medicare coverage) among infected older adults, the proportion of people with dual vulnerability might be higher in the U.S. population. Hence, it is of interest for future studies to use multiple sources of data to better represent populations who were infected with the disease. Second, based on cross-sectional data, this study cannot infer causality, although it seems plausible in the temporal sequence vulnerability and aging attitudes first, and emotional responses being the outcome. Third, there might be some confounding that was not controlled. For instance, we assumed that differences in political responses of states, territories, and counties contribute to the heterogeneity in inadequate healthcare utilization. However, residence information was lacking in this data. Lastly, the uncertainty in membership assignment was not considered in weighted regressions, despite bivariate analyses using a 3-step procedure supporting these findings.

## Conclusion

This study suggests that adults aged over 60 years presented three distinct patterns of vulnerability during the pandemic. About 67% of individuals were mildly vulnerable, and more than 30% of respondents had HV or DVs. Besides, individuals not eligible for Medicaid were more likely to have healthcare vulnerability, and Hispanics and non-Hispanic Blacks were more likely to have dual vulnerability. Meanwhile, adults below 65 years were prone to have healthcare vulnerability or dual vulnerability other than MV. While older adults from different vulnerability types had no significant difference in PAs, those with HV or dual vulnerability were likely to have more NAs. Besides, positive aging attitudes were associated with more positive and fewer NAs, and this salutary effect is more significant for individuals with dual vulnerability. Thus, we urge local governments and communities to keep a watchful eye on older adults during crises, with individuals having healthcare or dual vulnerability being prioritized. In addition, encouraging positive aging attitudes might be a critical approach for social services to promote the emotional well-being of older adults, especially those with dual vulnerability.

## Data Availability Statement

The data that support the findings of this study are available on request from the corresponding author.

## Ethics Statement

The studies involving human participants were reviewed and approved by Yale University Institutional Review Board. The patients/participants provided their verbal informed consent to participate in this study.

## Author Contributions

MF carried out the statistical analysis and drafted the manuscript. JG assisted with writing the article. XC critically reviewed this article. BH, FA, and MS participated in the revision of this manuscript. QZ designed the study and revised the manuscript. All authors have read and approved the manuscript.

## Funding

This study was supported by the National Social Science Fund of China (20VYJ030); the Guizhou Kong Xuetang Development Foundation; the U.S. PEPPER Center Scholar Award (P30AG021342) and an NIH/NIA grant (K01AG053408).

## Conflict of Interest

The authors declare that the research was conducted in the absence of any commercial or financial relationships that could be construed as a potential conflict of interest.

## Publisher's Note

All claims expressed in this article are solely those of the authors and do not necessarily represent those of their affiliated organizations, or those of the publisher, the editors and the reviewers. Any product that may be evaluated in this article, or claim that may be made by its manufacturer, is not guaranteed or endorsed by the publisher.

## Abbreviations

COVID-19: Coronavirus Disease
HRS: Health and Retirement Survey
I-PANAS-SF: International Positive and Negative Affect Schedule Short-form
ATOT: Attitudes toward own aging
PGC: Philadelphia Geriatric Center
PA: Positive Affect
NA: Negative Affect
LCA: Latent Class Analysis
AIC: Akaike Information Criterion
BIC: Bayesian Information Criterion
ssaBIC: sample size-adjusted BIC
LMRT: Lo-Mendell-Rueben Test (LMRT)
MV: Mild vulnerability
HV: Healthcare use vulnerability
DVs: Dual vulnerability in healthcare use and financial sustainment.
