# Supplementary material for: American Older Adults in COVID-19 Times: Vulnerability Types, Aging Attitudes, and Emotional Responses
Source: Front Public Health. 2022 Feb 8;9:778084. doi: 10.3389/fpubh.2021.778084 (PMC8860986; doi:10.3389/fpubh.2021.778084)
Supplement: Supplementary file 1 [file Table_1.DOCX]

**TableS1.** Bivariate analyses on the associations between vulnerability type and socioeconomic/health variables using 3-step regression in Mplus (reference: MV)

|  | **HV** | | **DVs** | |
| --- | --- | --- | --- | --- |
|  | **Estimate (S.E)** | **P value** | **Estimate (S.E.)** | **P value** |
| **Sex** (0=female, 1=male) | -0.164 (0.076) | 0.030* | 0.138 (0.076) | 0.070 |
| **Marital status** (0=uncoupled, 1=married/partnered) | -0.158 (0.175) | 0.367 | -1.820 (0.829) | 0.028* |
| **Education** (0=middle school below, 1=middle school above) | -2.507 (1.925) | 0.193 | -0.281 (0.180) | 0.119 |
| **Age** (0=65 years below, 1=65 years and above) | 0.525 (0.290) | 0.070 | -1.588 (0.492) | 0.001*** |
| **Difficulty in daily activities** (0=none, 1=one and more) | 0.663 (0.188) | <0.001*** | 2.438 (1.723) | 0.157 |
| **Self-rate health** (0=relatively poor, 1=relatively good) | 0.205 (0.557) | 0.713 | 0.224 (0.688) | 0.745 |
| **Medicaid** (0=not eligible, 1=eligible) | -0.019 (0.055) | 0.732 | 0.127 (0.048) | 0.008** |
| **Affective profile** |  |  |  |  |
| Self-fulfilling profile | -1.099 (0.731) | 0.133 | -2.039 (0.727) | 0.005** |
| High-affective profile | 0.397 (0.782) | 0.612 | 0.148 (0.780) | 0.850 |
| Low-affective profile | -1.380 (0.669) | 0.039* | -1.559 (0.604) | 0.010** |
| Self-destructive profile | 1.454 (0.588) | 0.013* | 1.692 (0.581) | 0.004** |
| **Race** |  |  |  |  |
| Non-Hispanic White | -0.238 (0.194) | 0.220 | -1.692(0.581) | 0.004** |
| Hispanic | 0.191 (0.188) | 0.309 | -3.949(4.582) | 0.389 |
| Non-Hispanic Black | 0.113 (0.273) | 0.679 | 1.701 (0.479) | <0.001*** |
| Others | -0.047 (0.239) | 0.843 | 0.916 (0.488) | 0.060 |
| **Household wealth** |  |  |  |  |
| Relatively poor | 0.008 (0.177) | 0.962 | 0.319 (0.502) | 0.525 |
| Medium level | -0.123 (0.152) | 0.418 | -0.007 (0.105) | 0.949 |
| Relatively rich | -0.034 (0.036) | 0.353 | -0.045 (0.039) | 0.251 |

**Notes:**

**Abbreviations**: MV: Mild vulnerability; HV: Healthcare use vulnerability; DVs: Dual vulnerability in healthcare use and financial sustainment; S.E., standard error. * P<0.05, **P<0.01, ***P<0.001.
